# YAP/TAZ Signalling Controls Epidermal Keratinocyte Fate

**DOI:** 10.3390/ijms252312903

**Published:** 2024-11-30

**Authors:** Maria D. Pankratova, Andrei A. Riabinin, Elizaveta A. Butova, Arseniy V. Selivanovskiy, Elena I. Morgun, Sergey V. Ulianov, Ekaterina A. Vorotelyak, Ekaterina P. Kalabusheva

**Affiliations:** 1 Cell Biology Laboratory, Koltzov Institute of Developmental Biology, Russian Academy of Sciences, 119334 Moscow, Russia; masha.pankratova25@bk.ru (M.D.P.); andrey951233@mail.ru (A.A.R.); butova.liza@gmail.com (E.A.B.); lady.morgun2016@yandex.ru (E.I.M.); kalabusheva.e@gmail.com (E.P.K.); 2 Laboratory of Structural-Functional Organization of Chromosomes, Institute of Gene Biology, Russian Academy of Sciences, 119334 Moscow, Russia; arseniy3001@mail.ru (A.V.S.); sergey.v.ulyanov@gmail.com (S.V.U.); 3 Department of Molecular Biology, Faculty of Biology, M.V. Lomonosov Moscow State University, 119234 Moscow, Russia

**Keywords:** YAP, TAZ, Hippo, keratinocytes, epidermis, skin, signalling, transcription

## Abstract

The paralogues Yes-associated protein (YAP) and transcriptional coactivator with PDZ-binding motif (TAZ) control cell proliferation and cell fate determination from embryogenesis to ageing. In the skin epidermis, these proteins are involved in both homeostatic cell renewal and injury-induced regeneration and also drive carcinogenesis and other pathologies. YAP and TAZ are usually considered downstream of the Hippo pathway. However, they are the central integrating link for the signalling microenvironment since they are involved in the interplay with signalling cascades induced by growth factors, cytokines, and physical parameters of the extracellular matrix. In this review, we summarise the evidence on how YAP and TAZ are activated in epidermal keratinocytes; how YAP/TAZ-mediated signalling cooperates with other signalling molecules at the plasma membrane, cytoplasmic, and nuclear levels; and how YAP/TAZ ultimately controls transcription programmes, defining epidermal cell fate.

## 1. Introduction

The skin epidermis is a multilayered and rapidly self-renewing epithelium that protects an organism from dehydration, pathogens, chemicals and physical damage [1]. Keratinocyte (KC) turnover starts in the basal layer, the deepest layer in the epidermis, located at the basal lamina. The basal layer contains epidermal stem cells; their quiescence and cell cycle are regulated by external signals from the stem cell niche that result in the production of amplifying progenitors and differentiated cells. During differentiation, KCs move upward and sequentially form spinous, granular, and cornified layers [2,3,4]. Cells in each epidermal layer have distinctive properties dictated by transcriptional programmes leading to the expression of specific layer markers.

Mitotically active basal KCs are characterised by the expression of keratins 5 (KRT5), 14 (KRT14), and 15 (KRT15). Basal stem cells are also distinguished by high expression of adhesion proteins. Basal KCs are attached to the basal lamina through hemidesmosomes and focal adhesions containing integrins. KCs contact each other through adhesive junctions (AJs) and desmosomes, which connect actin cytoskeletons and keratin filaments, respectively. After leaving the basal layer, cells downregulate the expression of basal cell markers and activate the expression of suprabasal keratins 1 (KRT1) and 10 (KRT10), as well as keratin 9 (KRT9) in the palmoplantar epidermis and 2e (KRT2e) in thickened sites. In the granular layer, even more differentiated KCs start synthesising keratohyalin granules. From the upper spinous to the cornified layer, the cornified cell envelope starts assembling and developing a physical barrier. This process involves structural proteins such as loricrin cornified envelope precursor protein (LORICRIN), involucrin (IVL), and trichohyalin (TCHH). Finally, the terminally differentiated KCs undergo an ‘epidermal programmed cell death’ and desquamate [4,5,6].

Skin derivates include hair follicles (HFs), sebaceous glands, and sweat glands. Throughout life, the HFs undergo cyclic changes that are categorised into stages: anagen (growth), catagen (regression), and telogen (resting). Catagen results in the degeneration of the lower part of the HF. During anagen, HFs regenerate their structure. HFs contain rapidly dividing matrix cells in hair bulbs that undergo differentiation into the hair shaft and inner root sheath (IRS). During the hair cycle, the matrix cell population is replenished by descendants from the stem cell niche located in the bulge area of the outer root sheath (ORS) [7,8].

Therefore, the epidermal cell phenotype could be characterised by belonging to HFs or the interfollicular epidermis (IFE) and by the level of differentiation, which corresponds to cell proliferative ability. During skin injury or in a proinflammatory microenvironment, KCs can undergo partial epithelial–mesenchymal transition (EMT) and upregulate several markers, including keratins 6 (KRT6), 16 (KRT16), and 17 (KRT17), which are not normally expressed in the IFE [9]. The paralogues Yes-associated protein (YAP) and transcriptional coactivator with PDZ-binding motif (TAZ) could participate in the regulation of KCs, acquiring a particular cell phenotype.

YAP and TAZ are typically described as members of the Hippo pathway. Hippo is an evolutionarily highly conserved signalling pathway first identified in *Drosophila melanogaster*. The mammalian Hippo pathway consists of mammalian STE20-like kinase 1/2 (MST1/2), large tumour suppressor kinases 1/2 (LATS1/2), and two scaffold proteins, Salvador homologue 1 (SAV1) and MOB (monopolar spindle-one-binder proteins) kinase activator 1A and 1B (MOB1A/B). The activation of this signalling pathway results in the phosphorylation of YAP and TAZ. Phosphorylated YAP/TAZ are inactivated, sequestered in membrane-associated protein complexes or undergo proteasomal degradation. Active non-phosphorylated YAP/TAZ can translocate to the nucleus, where they function as transcriptional regulators. They do not directly bind DNA regulatory elements and upregulate the transcriptional activity via transcription factors (TFs) predominantly from the TEA domain (TEAD) family [10,11,12].

The key role of the Hippo pathway, mediated YAP/TAZ activity, was revealed in the cell differentiation of epithelia in various organs [13,14,15,16,17]. Activated YAP and TAZ are essential for proper epidermal development and self-renewal [18,19]. Along with the Hippo pathway, YAP and TAZ activation is regulated by mechanotransduction and signalling pathways that define KCs’ stemness, proliferation, differentiation and injury response (e.g., WNT/β-catenin [CTNNB1], transforming growth factor beta [TGFβ], and epidermal growth factor [EGF] receptor [EGFR]/phosphoinositide 3-kinase [PI3K]) [14,20,21,22,23,24,25]. Due to YAP and TAZ being involved in normal skin epidermis maintenance [26] and the progression of many diseases [27,28], YAP/TAZ activation mechanisms are of particular interest and require comprehensive investigation.

The growing number of review articles on YAP/TAZ involvement in cell differentiation [29,30,31], cancer progression [32,33], organogenesis [34,35,36,37], and ageing [38,39] indicates that evidence of YAP/TAZ functions is rapidly accumulating and must be summarised and analysed. In this review, we discuss the mechanisms of YAP and TAZ activation in the skin epidermis and the transcription programme regulated by YAP and TAZ activity in epidermal KCs.

## 2. YAP/TAZ Distribution in Normal and Diseased Epidermis

Nuclear YAP appears when the epidermis comprises a single layer of KCs during embryogenesis [18,19,40]. During further development and in adult skin, YAP and TAZ are found in the nuclei of basal KCs and the cytoplasm in the suprabasal layers [14,19]. In aged skin, the number of cells with active YAP decreases along with the proliferation rate [14,40]. KCs with active nuclear YAP exhibit increased expression of proliferation markers and tumour protein p63 (TP63), a key TF that determines epidermal growth [41].

HF development begins with reduced *YAP* expression in epidermal placodes; however, YAP remains in an active nuclear state [40]. During the subsequent stages of HF development and hair cycle, YAP is distributed in the nuclei in proliferative HF structures—the hair matrix and ORS—while it remains in the cytoplasm in differentiating IRS and hair shaft cells [21,26,42].

During wound healing, *YAP* and *TAZ* expression is upregulated in the epidermis and dermal fibroblasts [14,19]. In contrast, ultraviolet radiation inhibits cell proliferation and decreases the expression of *YAP* and its targets [43,44]. In diseased skin, YAP/TAZ overactivation correlates with a hyperproliferative phenotype: psoriasis [45,46,47], lichen planus [48], and several skin cancers, including basal cell carcinoma (BCC), squamous cell carcinoma (SCC), and melanoma [49,50,51,52,53]. Human papillomavirus progression requires YAP/TAZ nuclear recruitment to upregulate KC proliferation [54,55]. In contrast, inherited epidermal abnormalities, such as epidermolysis bullosa, are characterised by defective, low-proliferation KCs, and predominantly cytoplasmic YAP localisation in the epidermis [41].

The correlation between nuclear YAP localisation and proliferation rate is also observed in cultured epidermal KCs. KCs with active YAP signalling exhibit elevated integrin and *p63* expression, rapid adhesion, and high colony formation efficiency [19,41,56]. *YAP* and *TAZ* expression decreases during KCs passaging [41,57]. In growing KC colonies, nuclear YAP is localised specifically at the colony edges, while interior cells retain only cytoplasmic YAP [19,41]. Establishing this pattern is essential for contact inhibition: the suppression of proliferation upon increasing cell density. Preserving YAP in the cytoplasm is ensured by cell contact stabilisation mediated by E-cadherin and associated 14-3-3 proteins [41,58].

Culture conditions and the application of substrates with specific properties allow the exploration of the mechanosensory nature of YAP/TAZ signalling. The application of ‘soft’ substrates (with an elastic modulus of up to 4 kPa) prevents YAP activation, while ‘stiff’ substrates induce YAP nuclear translocation [25,45,59,60]. Therefore, soft substrates stimulate the expression of KC differentiation markers [59]. Like contact inhibition, mechanosensing relies on cell-contact proteins and associated cytoskeletal filaments. This mechanism likely contributes to the YAP activation pattern in the epidermis, reflecting the varying stiffness experienced by basal and more differentiated layers [45].

In summary, nuclear YAP and TAZ are features of proliferating KCs, while cytoplasmic YAP and TAZ are specific to early differentiating cells.

## 3. YAP/TAZ Signalling Modulation and Its Influence on Epidermal Cell Fate

### 3.1. Approaches to Modulating YAP/TAZ Activity

YAP/TAZ signalling is usually enhanced or inhibited to understand YAP/TAZ regulation. YAP/TAZ can be enhanced by overexpression or overactivation. Overexpression increases the amount of YAP and TAZ protein and does not affect the regulation of their activity. Overexpression is primarily used for primary KC cultures [41,57].

Overactivation leads YAP/TAZ to accumulate in the nucleus, where they can regulate gene expression. Constitutively active YAP isoforms are created via genetic modification at serine residues, rendering them resistant to LATS-mediated cytoplasmic sequestration. Mutating serine 127 to alanine (S127A) [18,40,57] or applying YAP lacking serine residues (YAP2-5SA) [14] increases nuclear YAP levels. Overactivated YAP is suitable for animal models, while inserting the constitutively active YAP isoform into primary KCs in culture results in their senescence and death [41]. The YAP2-5SA-ΔC variant also lacks the C-terminal region from Q281, including a transactivation domain. This mutation prevents YAP-driven gene expression in vitro. This mutant protein was expected to block endogenous YAP function and disrupt epidermal homeostasis. However, transgenic mouse lines exhibit typical YAP overactivation phenotypes [21,42]. To ensure skin-specific expression, *KRT5* and *KRT14* gene promoters were used with or without an inducible system [40,42,52].

Another way to obtain mice with YAP overactivation is by targeting the Hippo pathway. MOB1A/B deficiency strongly activated endogenous YAP1 in mice [61,62]. In addition, 14-3-3 proteins are critical for excluding phosphorylated YAP1 from nuclei; the skin of *Er/Er* mice carrying the repeated epilation mutation (*Er*) of 14-3-3σ protein failed to retain YAP1 in the cytoplasm [63]. LATS kinase inhibitors activate YAP/TAZ signalling by preventing their LATS-dependent phosphorylation and consequent degradation [64]. A small-molecule agent, PY-60, activates YAP-driven transcription by blocking the Hippo pathway scaffolding protein annexin A2 (ANXA2) [65,66].

Mice with skin-specific *Krt5/14-Cre* or *CreERT* were crossed with YAP/TAZ-floxed mice to create a double conditional knockout (KO) [14,18,67]. To knockdown (KD) YAP/TAZ in the epidermis, cells were infected with a lentiviral vector carrying YAP- or TAZ-specific short hairpin RNA (shRNA) or small interfering RNA (siRNA) [40,41,68]. A genetically encoded dominant-negative protein (TEADi) was designed to inhibit TEAD-dependent YAP/TAZ expression regulation. It contains TEAD-interacting domains that bind to TEADs and prevent their interaction with transcriptional coactivators [68]. Serine 94 in the human YAP and serine 79 in the mouse orthologue are essential for YAP’s interaction with TEADs [59]; changing them to alanine inhibited YAP/TEAD communication and suppressed YAP activity in genetically modified animals [18]. Finally, small-molecule inhibitors of YAP/TAZ signalling have been discovered. Verteporfin (VP) blocks the interaction of YAP with TEAD, preventing the transcriptional activation of YAP downstream targets [69]. VP also induces sequestration of YAP in the cytoplasm by increasing levels of 14-3-3σ and its subsequent proteasomal degradation [70,71]. Peptide 17 is a more selective inhibitor; it similarly disrupts the binding of YAP and TEAD but does not prevent the translocation of YAP into the nucleus [72,73]. The Src family kinase inhibitor dasatinib is known to strongly inhibit YAP activation [52]. Simvastatin has been identified as an efficient agent for inhibiting TAZ [53,67].

### 3.2. Alterations in KC Marker Expression Under YAP/TAZ Modulation

YAP/TAZ overactivation or inhibition affects KC-specific marker expression and proliferation and the structure of the skin and its derivatives.

Doxycycline-mediated expression of active YAP (S127A) under the *KRT14* promoter significantly altered mouse skin structure [40]. After induction, epidermal development demonstrated hypertrophy, hyperproliferation, and increased basal marker expression, whereas expression of the differentiation markers *KRT10* and *LORICRIN* was mostly absent, even during embryogenesis. HF structure was disrupted, although the hair-specific TFs, controlling the specification of the HF’s layered structure, were preserved [18,40]. YAP2-5SA-ΔC mice exhibit milder deviations that gradually develop during the postnatal period. From four to five weeks of age, the skin becomes dry and fragile, with lesions and hair loss. At postnatal day 85 (P85), all epidermal layers are thickened, leading to hypertrophy and hyperkeratinisation [42].

The *Yap* KO mouse model exhibited thinner and fragile skin with a loss of epidermal tissue on limbs and abnormal epidermal architecture and died at the embryonic stage or shortly after birth. Histological analysis revealed a hypoplastic basal layer with abnormal cell morphology, decreased progenitor cells, and a reduced cornified layer [18]. Like with *YAP* overactivation, YAP/TAZ double KO mice demonstrate progressive alopecia in neonates or after two weeks post-tamoxifen injection in adults [14].

**Figure 1:**
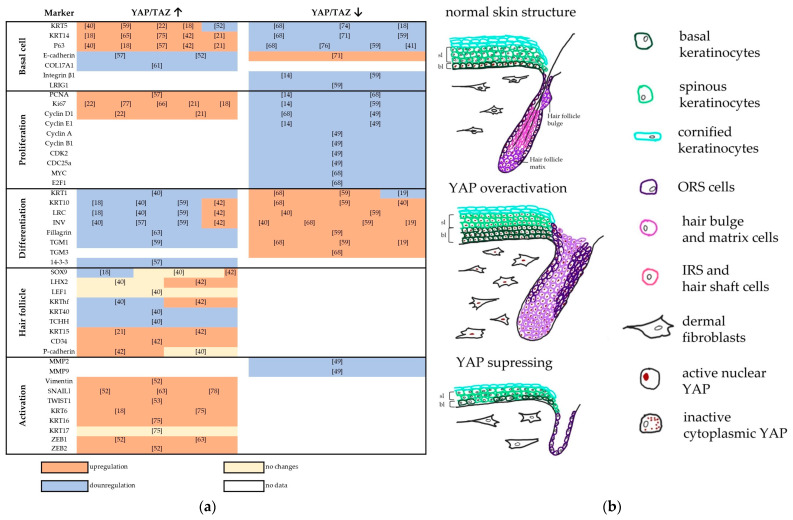
(**a**) A table summarising the data on the increase or decrease in various markers under YAP overactivation and suppression. (**b**) Top panel: in normal skin, YAP is localised in nuclei in the basal layer of the epidermis and is detected in the cytoplasm in differentiated layers. In mature HFs, YAP is mainly cytoplasmic in differentiated cells of the IRS and hair shaft and is retained in the nucleus in the hair matrix and ORS. Middle panel: YAP overactivation results in abnormal HF structure, widening of the basal layer of the epidermis, and disruption of terminal differentiation, as well as a fibrotic state of the dermis with fibroblast hyperproliferation and increased collagen production. Bottom panel: YAP suppression disrupts the HF cycle and forms a thin epidermis with abnormal stratification. Abbreviations: BL, basal layer; IRS, inner root sheath; SL, spinous layer; ORS, outer root sheath [14,18,19,21,22,40,41,42,49,52,53,57,59,61,63,65,66,68,71,74,75,76,77,78].

Below, we summarise the published evidence on the upregulation and downregulation of KC-specific markers under YAP overactivation and suppression conditions (Figure 1).

#### 3.2.1. Proliferation

The most significant changes are related to the proliferation rate. Proliferation marker expression in the epidermis of both YAP (S127A) and YAP2-5SA-ΔC mice indicates a substantial increase in the number of positive cells in the basal layer and an extension into the suprabasal layers [18,21,22,40,42,75,79]. The same effects were observed under treatment with a small-molecule YAP activator [65]. YAP/TAZ KO mice exhibited a significant decrease in proliferating basal cells [14,18].

Cultured KCs are also sensitive to YAP signalling modulation. *YAP* overexpression enhanced proliferation, immortalisation, senescence escape, and clonal evolution blockage in primary human KCs [41,57]. Indeed, YAP2-5SA- and YAP (S127A)-transduced KCs increased in size, expressed β-galactosidase, and overexpressed cyclin-dependent kinase inhibitor 2A (*CDKN2A/p16^INK4a^*) and tumour protein p53 (*TP53*), indicating cell senescence [41]. Overexpressed *YAP* escapes the E-cadherin-mediated contact inhibition of proliferation [58]. In contrast, YAP and TAZ KD results in cell differentiation, inhibited proliferation, cell cycle arrest, and apoptosis promotion in primary and immortalised KCs [41,46,49,68]. Therefore, in vivo and in vitro evidence indicates a regulatory role for YAP and a supporting role for TAZ in cell proliferation.

#### 3.2.2. Differentiation

YAP overactivation-induced skin hypertrophy results from increasing numbers of KRT5/14- and p63-positive basal cells and their propagation into suprabasal layers [18,40,42]. The thickness of superior layers depends on YAP modification. In the presence of the YAP2-5SA-∆C mutant, the IFE exhibits an expansion of the spinous and granular layers, expressing KRT10 and IVL, respectively, and thickening of the cornified layer, indicating hyperkeratinisation [42]. In contrast, in the presence of the YAP (S127A) mutant, there is a significant thinning or absence of differentiated layers expressing KRT1/10 and LORICRIN in the epidermis [18,40]. These differences are related to mouse age and the intensity of skin abnormality progression that appeared even in embryogenesis for YAP (S127A) mice but several weeks after birth for YAP2-5SA-∆C mice.

*Yap* KO and TEADi provoked aberrant distribution of KRT5/14 in the basal layer and the appearance of differentiated KRT1/10 cells in the basal lamina of mouse skin. All the layers became thinner, and the number of KRT10- and LORICRIN-positive cells decreased [18,68]. In contrast, epidermis structure did not differ between *Taz* KO and control mice [67]. Thus, YAP is the predominant paralog in epidermal self-renewal in skin homeostasis.

In cultured KCs, YAP activation is responsible for differentiation inhibition [45,57,59,68]. The analysis of differentiation markers revealed that YAP maintains a high proliferation rate by preserving the progenitor features of KCs but does not induce cell dedifferentiation and re-entry into the cell cycle of differentiated cells [41].

Collagen XVII (COL17A1), a component of the hemidesmosomes, is involved in cell turnover in the basal layer: its differential expression between the descendants of dividing basal KCs defines the cell’s persistence in the basal layer or movement to the suprabasal layers [80]. Increased *COL17A1* expression corresponds to a fraction of slowly dividing basal KCs with stemness properties [81]. Basal KCs in *Mob1a*/*b*-deficient (YAP1-activated) mouse skin lose *COL17A1* expression. In vitro, *Mob1a*/*b*-deficient cells demonstrate impaired adhesive properties and are eliminated from the substrate by wild-type cells [61]. Therefore, it can be assumed that rapidly dividing cells with nuclear YAP are transiently amplifying progenitors rather than stem cells.

#### 3.2.3. Hair Follicles

Both YAP activation and suppression lead to hair loss but via different mechanisms. YAP deletion or TEAD-binding inhibition reduces the proliferative cell fraction in HFs and the IFE, resulting in progressive hair loss [14,68]. YAP (S127A) mutants with pronounced alterations in embryo epidermis exhibit abnormalities is HF structure, but not in HF localisation [40]. The HFs of both YAP (S127A) and YAP2-5SA-ΔC mice strains acquire disorganised structure and predominantly consist of proliferating cell mass positive for bulge and hair matrix markers [40,42]. YAP overactivation regulates HF progenitor cell proliferation and prevents their subsequent differentiation; however, it does not influence cell fate choice to become either HF or IFE based on preserving the expression of TFs specific to the different HF layers [40,42].

HF formation also appears after wounding in mouse skin through HF neogenesis [82]. In this case, the number of HFs was greater under YAP overactivation; they similarly exhibited abnormal morphology [83]. This highlights the common action of YAP with other signalling pathways involved in HF regeneration.

#### 3.2.4. Wound Healing

During skin regeneration, epidermal cells acquire an injured phenotype and undergo partial EMT, which is necessary for KC migration and proliferation. Both YAP and TAZ guide the epidermal restoration after injury. YAP/TAZ KD and KO delayed wound healing in mouse skin [14,84], while their activation promoted wound closure [65,66]. Although *Yap* overexpression does not initiate EMT [43], YAP and TAZ stimulate cell migration [36,38], proliferation [9] and prevent apoptosis [67,77].

YAP/TAZ also activate the expression of genes involved in inflammation. Direct targets of YAP/TAZ include cysteine-rich angiogenic inducer 61 (CYR61, currently called cellular communication network factor 1 [CCN1]), connective tissue growth factor (CTGF; currently called cellular communication network factor 2 [CCN2]), and TGFβ pathway members [50,84]. CCN1 is involved in KC injury response [43,85,86], and CCN2 is required for re-epithelisation, proliferation, and tissue remodelling [87,88]. YAP1 stimulates KCs to produce the colony-stimulating factor 1 (CSF1) in the wound microenvironment, which supports macrophage attraction in a paracrine manner [89].

YAP and TAZ activity must be transient for successful tissue regeneration. Chronic inflammation or signalling abnormalities prolong the timing of YAP and TAZ activity and result in their nuclear accumulation, which leads to SCC formation [52], EMT [52,53,90] and increased proinflammatory cytokine and growth factor levels [87,88,91,92]. Under YAP overactivation, mouse skin reproduced a psoriatic phenotype with hypertrophic epidermis that was positive for KC injury-associated markers KRT6/16 and a fibrotic state in the dermis with fibroblast hyperproliferation and increased collagen production [75,79,83].

## 4. Mechanisms of YAP/TAZ Activation in the Epidermis

### 4.1. Hippo-Dependent YAP/TAZ Activation

The mechanism of YAP/TAZ nuclear shuttling is context-dependent and differs between organs, tissues, and cell types; it involves a group of regulatory elements controlling YAP and TAZ activity directly or via the Hippo pathway. Hippo signalling plays a key role in the differentiation and specification of simple and pseudostratified epithelia [12,36,93,94,95]. Here, we discuss the particular mechanisms of YAP/TAZ activation in stratified cornifying epidermis.

Under Hippo activation, active MST1/2 phosphorylates SAV1 and MOB1/2, which assist MST1/2 in recruiting and phosphorylating LATS1/2. Activated LATS1/2 serine/threonine kinases phosphorylate YAP and TAZ on their serine residues. In humans, inhibition includes phosphorylation of S127 and S381 in YAP and S89 and S311 in TAZ. YAP-S127 and TAZ-S89 phosphorylation is required for 14-3-3 protein binding to establish the molecular complex for cytoplasmic sequestration and subsequent inactivation. S381/S311 phosphorylation of YAP/TAZ, respectively, initiates proteasomal degradation. Dephosphorylases revert active YAP/TAZ conformation and signalling activity. The canonical Hippo pathway has been discussed in detail in several recent reviews [28,96].

Hippo pathway suppression promotes YAP activation during embryonic skin development [40,61] or until confluence is established in KC cultures [97,98], but not after achieving contact inhibition [18,19]. Unlike other epithelial types, Hippo activity is not controlled by cell polarity protein complexes in the epidermis [58]. YAP and TAZ cytoplasmic states are maintained primarily by cell–cell and cell–matrix contacts [97,98]. Junction-associated intracellular proteins 14-3-3 and α-catenin sequester phosphorylated YAP and TAZ in the cytoplasm [63,98,99,100]. Disrupting the α-catenin/14-3-3σ/YAP complex allows YAP to interact with the catalytic subunit of protein phosphatase 2A (PP2AC), triggering its activation [18].

The E-cadherin/AJ and actomyosin complex, including F-actin, myosin II, actin-related proteins 2/3 (ARP2/3), and myosin light chain kinases (either myosin light chain kinase [MLCK] or Rho-associated kinase [ROCK]), mediates mechanosensory YAP/TAZ activation [45,98,101]. This process also involves desmosomal proteins desmoglein 3 (DSG3), adaptor plakophilins (PKPs) [60,97,102], and keratin filaments [103] to create the membrane YAP/TAZ-sequestration complex. YAP/TAZ nuclear shuttling appears under inflammation-induced AJ disassembly [101] or changes in matrix stiffness [59,98,104]. YAP/TAZ is mechanosensory-regulated even at the nuclear level. Nuclear F-actin-dependent binding of YAP and TAZ by the SWI/SNF chromatin-remodelling complex via the AT-rich interaction domain 1A (ARID1A) subunit prevents their interaction with TEADs [105].

Several studies suggest a key role of cell–matrix contacts and associated signalling pathways, such as integrin/Src and focal adhesion kinases (FAKs), in the activation of YAP/TAZ in the epidermis [14,90]. SRC proto-oncogene, non-receptor tyrosine kinase (Src) directly phosphorylates YAP at three binding sites on tyrosine residues (341, 357, and 394) that allow YAP activation and subsequent target gene expression [90]. Dysregulated integrin signalling appears in junctional epidermolysis bullosa due to mutations in laminin 332, COL17A1, or integrin α6β4, leading to YAP suppression as a part of its pathological development [14,41,81]. In skin homeostasis, integrin/Src-mediated YAP activation is negatively controlled by α-catenin [90] and Thy-1 cell surface antigen (THY1), a GPI-anchored protein that also functions as an important part of the AJ complex [83]. Besides epidermal self-renewal, integrin-mediated YAP activation via the FAK/Src [89] and PI3K/pyruvate dehydrogenase kinase 1 (PDK1) pathways [106,107] is involved in wound healing and cancer progression.

**Figure 2:**
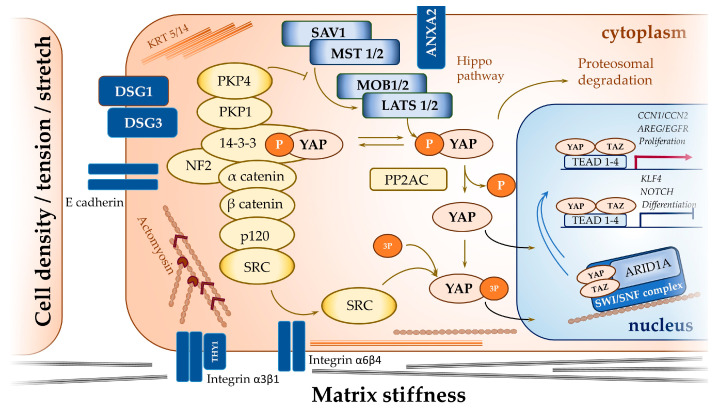
Mechanisms of YAP activation in KCs. The canonical Hippo signalling pathway via the kinases MST1/2 and LATS1/2 inactivate YAP/TAZ via serine phosphorylation to enhance retention in the cytoplasm and/or proteasomal degradation. Phosphorylated YAP is sequestered in the cytoplasm by a membrane protein complex that includes AJs, desmosome proteins, integrins, and their cytoplasmic adaptors. In the complex, α-catenin controls YAP/TAZ activity and phosphorylation by modulating their interaction with 14-3-3 proteins and PP2AC. DSG3 and its adaptor protein plakophilin 1 (PKP1) are also involved in the membrane complex sequestration of YAP. Plakophilin 4 (PKP4) may function as a recruiter of YAP to the junctional zone or as a mediator of the disruptor of the YAP/TAZ/SAV1/LATS1 complex. ANXA2 was identified as a Hippo pathway modulator that controls YAP phosphorylation. Keratin filaments (KRT4/15) also control YAP activation through 14-3-3σ binding. Stabilisation of F-actin with functioning myosin II and ARP2/3 is required for stiffness-induced nuclear localisation of YAP/TAZ. Integrin/Src signalling contributes to the nuclear localisation of YAP, as Src can directly phosphorylate YAP at the three binding sites, activating it. Src activation mediated by integrin α6β4 negatively regulates α-catenin, whereas integrinα3β1/Src signalling is inhibited by their interaction with THY1. Additionally, YAP/TAZ activity is regulated at the nuclear level. Under mild mechanical stress in the nucleus, YAP/TAZ are sequestered in the pool of SWI/SNF chromatin remodelling complexes containing ARID1A, which prevents YAP/TAZ from binding to TEADs.

Most of the mechanisms of YAP activation in KCs are summarised in Figure 2.

### 4.2. YAP/TAZ Interaction with Non-Hippo Signalling Pathway Epidermal KCs

YAP/TAZ interacting with other signalling pathways that participate in epithelial morphogenesis [12] and skin pathology, including ageing [39], fibrosis [108,109], and cancer [28], has been accurately discussed in recent reviews. In addition, several articles have focused on particular signalling pathways [110,111,112] and YAP/TAZ involvement in the signalling microenvironment [113,114,115]. Here, we summarise the YAP/TAZ interplay in KCs.

#### 4.2.1. WNT/β-Catenin

The canonical WNT pathway activates β-catenin to regulate epidermal morphogenesis, HF formation and cycling, epidermal self-renewal, and stem cell pool maintenance [8,116,117,118] in KCs. YAP/TAZ and WNT/β-catenin interact in a mutually activating and suppressing manner. *YAP* and β-catenin expression and nuclear localisation patterns overlap within the epidermis. Increased proliferation in YAP2-5SA-ΔC hyperplastic skin and immortalised KCs (HaCaT cell line) is associated with canonical WNT/β-catenin activation via YAP-mediated Wnt family member 16 (*WNT16*) expression [21,22]. WNT/β-catenin drive skin abnormality progression induced by YAP overactivation [22,98].

The Hippo/YAP/TAZ and WNT/β-catenin pathways are connected at several levels. Firstly, YAP, TAZ, and β-catenin are sequestered in a common membrane complex that includes LDL receptor-related protein 6 (LRP6), axin 1 (AXIN1), glycogen synthase kinase 3 beta (GSK3β), and dishevelled [98,119,120,121]. YAP depletion significantly increased activated β-catenin via AJ complex destabilisation [68]. Several studies have proposed the existence of nuclear complexes that included YAP/TEAD interacting with LEF/TCF [122,123]. This observation highlights the common action of WNT and YAP in maintaining the undifferentiated and highly proliferative progenitor state.

#### 4.2.2. Sonic Hedgehog Signalling Molecule (SHH)

SHH and WNT/β-catenin are involved in HF development and regeneration; however, both pathways also drive skin tumour progression. SHH upregulation was accompanied by YAP and WNT/β-catenin activation in mouse and human BCC [74,79]. In turn, YAP promotes the expression of SHH GLI family zinc finger 2 (GLI2) by activating β-catenin [79]. SHH and WNT are involved in YAP-derived increases in HF number after wounding in mouse skin [82]. WNT/β-catenin, SHH, and YAP/TAZ support the proliferation of low-differentiation cells, which is essential in epidermal self-renewal and HF cycling. Overactivation of all three signalling pathways in mouse skin leads to highly similar abnormalities in epidermal structure, including epidermal thickening and HF disorganisation, indicating positive feedback and cumulative regulation [42,79,124].

#### 4.2.3. Notch

Unlike the WNT/β-catenin pathway, the Notch pathway induces epidermal cell commitment and differentiation [125]. YAP acts as a Notch antagonist. YAP activation upregulates the expression of Notch pathway inhibitors delta-like canonical Notch ligands 1 (*DLL1*) and 3 (*DLL3*) and NEDD4-like E3 ubiquitin-protein ligase (*NEDD4L*)*,* while cytoplasmic YAP sequestration results in Notch-mediated subsequent KC differentiation [59,126]. YAP/TAZ suppression of Notch activity maintained low differentiation of the hypertrophic epidermis in genetically modified mice [40,59] and BCC [74].

#### 4.2.4. EGF/EGFR

EGF stimulates KC proliferation in the homeostatic epidermis and promotes cell migration during wound healing. EGF/EGFR activity is required for YAP nuclear localisation in epidermal basal KCs [14,23]. The basic EGF/EGFR signalling pathway acts via the Ras/Raf/mitogen-activated protein kinase kinase (MEK)/extracellular signal-regulated kinase (ERK) cascade [127]; however, it recruits PI3K/protein kinase B (AKT) [14] and AXL receptor tyrosine kinase (AXL)-mediated Hippo suppression [23,128,129] for YAP activation in the epidermis. EGFR also regulates YAP-derived motility by targeting YAP/DSG3 interplay [102] and via the conjunction of the EGFR/PI3K and EGFR/Src signalling [130]. PDK1, a downstream member of the EGFR/PI3K pathway, was recently identified as a disruptor of the Hippo complex, which is involved in regulating YAP phosphorylation in KCs [14] but not in SCC cells [23,131].

The EGF/EGFR pathway is one of the most upregulated under YAP/TAZ activation. Amphiregulin (AREG), a direct target of YAP and TAZ, is an EGFR ligand that promotes extensive cell proliferation after UV damage [67] and in multiple skin diseases [46,47]. This observation indicates a positive feedback between YAP/TAZ and EGFR activation [132].

#### 4.2.5. Fibroblast Growth Factors (FGFs)

The interplay of FGF7/10 with WNTs regulates basal cell proliferation and subsequent epidermal stratification during development [133]. In adult skin, FGF10 produced by dermal fibroblasts binds fibroblast growth factor receptor 2 (FGFR2b) in KCs to stimulate ERK-mediated YAP activity to induce skin regeneration after injury [44]. FGFs cooperate with other signalling pathways during wound healing, including TGFβ, a well-known YAP/TAZ partner in skin regeneration [134]. FGFR/YAP/TAZ interplay requires in-depth future investigation because their cooperation has already been explored in breast and liver cancer [135,136].

#### 4.2.6. G-Protein Coupled Receptors (GPCRs)

GPCRs are a family of membrane proteins involved in key physiological skin functions in homeostasis and pathology. Agonist activation of GPCRs promotes the exchange of GTP with GDP from the guanine nucleotide-binding protein alpha subunit (Gα). GTP-bound Gα dissociates from the guanine nucleotide-binding protein beta/gamma complex (Gβγ), and Gα and Gβγ separately activate a series of downstream effectors. The interplay between GPCRs and YAP in the skin epidermis has been carefully described in a recent review [137]. Briefly, the G protein subunit alpha S (Gαs) and its effector protein kinase A (PKA) suppress YAP1 and the SHH pathway to control skin development and act as an antioncogene [138]. In contrast, GPCR-G protein subunit alpha i (Gαi) activation can increase KC proliferation and suppress differentiation, leading to epidermal hyperplasia via upregulation of YAP but not SHH [139]. Uridine 5′-diphosphate-specific G protein-coupled pyrimidinergic receptor P2Y6 (P2RY6) was found to be involved in activating proinflammatory responses, including YAP and WNT upregulation, and could be involved in skin regeneration [140].

#### 4.2.7. TGFβ/Bone Morphogenetic Protein (BMP)/SMAD

TGFβ receptor (TGFβR) signalling activation results in type I and type II receptor dimerisation and the phosphorylation of receptor-regulated SMADs (R-SMADs), which form a complex with SMAD family member 4 (SMAD4), translocate to the nucleus, and activate the transcription programme. SMAD family members 1/5/8 (SMAD1/5/8) are usually related to BMP ligands, while SMAD family members 2/3 (SMAD2/3) are associated with TGFβ. SMAD family member 7 (SMAD7) has mostly inhibitory functions [141].

TGFβs are a major group of inflammation drivers in skin regeneration [142]. The control of the expression of TGFβ pathway members by YAP/TAZ has been explored in genetically modified mouse models and cultured human KCs [75,84]. YAP and TAZ work with TGFβ1 by creating molecular complexes with SMAD2/3 [24,25] to promote EMT, wound healing and carcinogenesis [24,25,53].

In contrast, BMP/SMAD1/5/8 and SMAD7 are involved in epidermal and HF development during embryogenesis in crosstalk with WNTs and FGFs [133,143,144]. In adult skin, BMPs regulate KC differentiation in the IFE and cell specification during HF regeneration [145]. The possible interplay of BMP/YAP/TAZ in the epidermis is indicated by their upregulation in several cancer types [146,147]. SMAD7 is involved in stabilising E-cadherin/β-catenin [148] and can form complexes with YAP [148], thus preventing its activation and preserving it in the cytoplasm. However, SMAD7 is involved in upregulating KC proliferation in psoriasis and carcinogenesis, indicating more complicated signalling regulation [149,150]. As YAP overexpression/overactivation destabilises the balance between proliferation and differentiation in the epidermis and HFs, BMPs are expected to be downregulated or incapable of realising their target expression.

#### 4.2.8. Nuclear Factor Kappa B (NF-κB)

The NF-κB pathway integrates the extracellular inflammation signals and transmits them via phosphorylation of the IκB kinase (IKK) complex, activating proinflammatory gene expression [151]. During skin development and postnatal homeostasis, the NF-κB pathway is involved not only in injury-related responses but also in the differentiation processes of HF patterning and morphogenesis [152].

YAP and NF-κB mutually regulate each other’s activity in inflammation but not in a morphogenetic manner. YAP-mediated positive regulation of NF-κB/p65 was found in HaCaT cells [46], while interleukin 17A (IL17A) upregulates actin-related gene 1 (ACT1)/NF-κB and subsequent YAP activation, resulting in psoriatic-like alterations in KCs [47]. NF-κB is one of the most upregulated signals in psoriasis [153], as well as YAP and TAZ [154].

In conclusion, YAP/TAZ sustain a series of positive feedback loops with proinflammatory signalling pathways (TGFβ, EGF, and NF-kB), WNT/β-catenin, and SHH that are involved in preventing the differentiation and maintaining proliferation. YAP suppresses differentiation, while TAZ accompanies YAP in case of injury or disease. This highlights the multi-level control of YAP and TAZ activity in homeostatic skin.

## 5. YAP/TAZ/TEAD in the Nucleus

### 5.1. YAP and TAZ Structure

Human YAP and TAZ have high primary sequence similarity and, thus, several shared structural features, including domain structure. However, the differences in their structures can influence their activation and interaction mechanisms with TFs in the nucleus to regulate gene expression.

The C-terminal region of both proteins contains the coiled-coil (CC) domain, transactivation domain, and PDZ-binding domain [155,156]. The PDZ-binding domain is responsible for their sequestration by 14-3-3 proteins and thus involved in controlling YAP/TAZ in the cytoplasm [157,158]. The SRC homology 3 domain (SH3)-binding motif (amino acids PVKQPPPLAP) located between the WW and the CC domains is sufficient for interaction with several proteins, including the YES proto-oncogene (YES) and Src kinases [156]. Thus, it indicates mostly common activation mechanisms for YAP and TAZ.

The TEAD-binding domain (TBD) is located in the N-terminal region of both transcriptional coactivators and consists of α-helixes and the Ω-loop with a linker region and contacts the TEADs in the N-terminal region [159,160]. The linker is shorter in TAZ than in YAP since it lacks the PXXFP motif, which is significant for the YAP/TEAD interaction [160]. However, this fact allows TEAD interactions with two TAZ molecules and the formation of a heterotetramer instead of a heterodimer [155].

The WW domain is sufficient for the interactions of YAP and TAZ with other TFs, regulatory proteins, and chromatin remodelers. TAZ contains only one WW domain, while the number of WW domains in YAP depends on the isoform: YAP1 contains one WW domain, YAP2 contains two WW domains, and YAP2-L differs from YAP2 in that it contains 16 amino acid residues in the transcription activation domain [161]. The type of isoform was found to be significant in tumour development [162,163,164]; however, it has been poorly investigated in the epidermis.

Several YAP and TAZ domains feature an intrinsically disordered region (IDR), including the CC domain, transactivation domain, and small IDR in the YAP C-terminal region. These domains are involved in liquid–liquid phase separation (LLPS) and non-specific interactions with other IDR-containing TFs during protein condensation [165]. This finding underscores that YAP/TAZ/TEAD can interact specifically and non-specifically with other TFs, and the nature of these interactions may influence the realisation of the cell transcription programme.

### 5.2. YAP/TAZ-Mediated Transcription Regulation

YAP and TAZ primarily interact with TFs of TEAD family members in the nucleus. All four members of the TEAD family are paralogues and have the same domain structure. Their N terminal region contains a variable region and DNA-binding TEA/ATTS domain. The TEA domain binds to DNA elements and TFs necessary for target transcription activation [166]. The C-terminal transactivation YAP binding domain (YBD) recruits transcription coactivators (YAP, TAZ, and vestigial-like family members 1–4 [VGLL1/2/3/4]) [167,168]. The TEA domain and YBD are separated by a proline-rich domain. Differences in the last two domain structures are presumably responsible for TEAD functional specialisation [169].

Complexes of YAP and TAZ with TEA domain transcription factor 1–4 (TEAD1/2/3/4) have different functions in skin development, homeostasis, and tumorigenesis [170]. TEAD1 is the predominant paralog expressed in epidermal KCs [171]. Along with TEAD3, it is involved in KC self-renewal and proliferation [19,172]. TEAD1 is also detected in various cancer types, including SCC [173,174,175]. *TEAD4* expression correlates with KC activation and EMT and, thus, promotes psoriasis and tumour formation [176,177,178,179,180]. TEAD2 appears to play a critical role during development and neural tube closure [181]. Elevated *TEAD2* expression has also been associated with cancer progression and tumour drug resistance [182,183].

**Figure 3:**
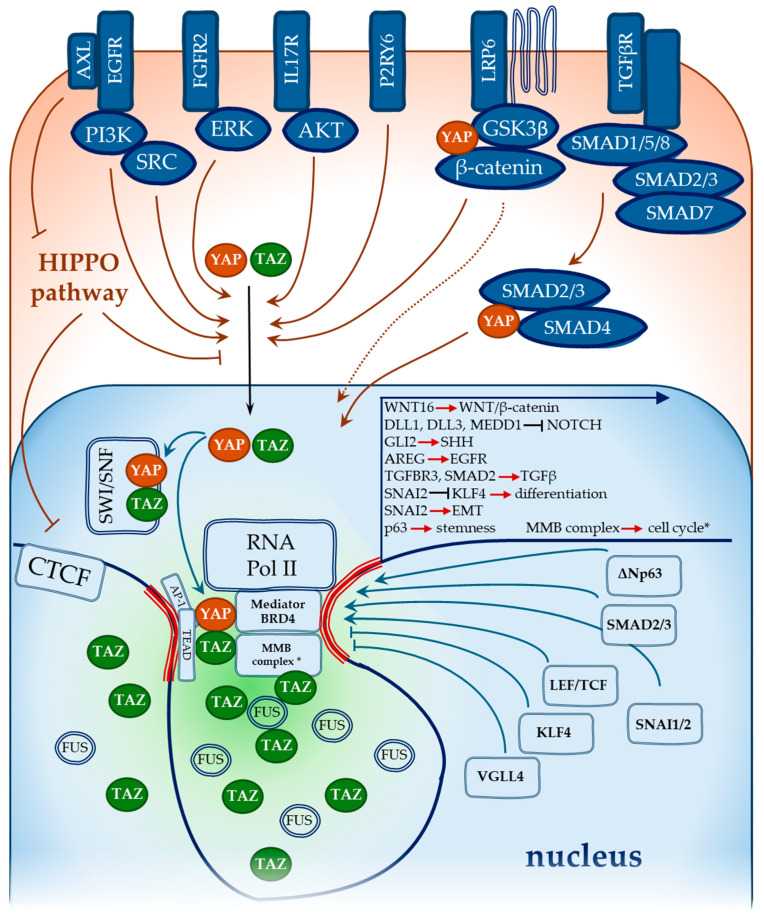
YAP and TAZ interactions with signalling pathways and TFs. EGFR, FGFR2, IL17R, and P2RY6 drive YAP and TAZ activation. WNT pathway activation disrupts the cytoplasmic YAP sequestration complex, resulting in the release of YAP and β-catenin and their nuclear translocation. TGFBR activation transduces the signal to the SMAD2/3 complex, which interacts with YAP/TAZ and binds SMAD4 for nuclear shuttling. SMAD7 is a component of the cytoplasmic protein complex that suppresses YAP activity. In the nucleus, YAP and TAZ are recruited to distant regulatory elements, enhancers and super-enhancers, in association with TEADs and AP-1. YAP and TAZ recruit BRD4 and the Mediator complex to promote transcription. YAP/TAZ-mediated engagement of the MMB complex initiates chromatin looping and transcription of cell cycle-related genes (*). Besides the MMB complex, YAP and TAZ could be involved in chromatin remodelling via association with the SWI/SNF complex and Hippo-mediated suppression of CTCF activity. LEF/TCF, SNAI1/2, SMAD2/3, and ΔNp63 can form transcription complexes with YAP/TAZ, while VGLL4 and KLF4 suppress YAP/TAZ-mediated transcription. TAZ can form nuclear condensates via LLPS. The FUS protein associates with TAZ in nuclear condensates, maintaining their liquid state and thus facilitating TAZ-mediated transcriptional regulation.

YAP/TAZ/TEAD bind mostly distant regulatory sequences (enhancers and super-enhancers) regulating target gene expression [184]. To drive the transcription, the YAP/TAZ/TEAD complex recruits bromodomain-containing protein 4 (BRD4) [185] and the Mediator complex to promote cyclin-dependent kinase 9 (CDK9)-dependent transcriptional activity [186]. In addition, YAP associates with the MYB proto-oncogene-like 2 (MYBL2/B-MYB) and MuvB TF complex, leading to the formation of the MMB complex, chromatin looping, and the induction of target gene expression, particularly cell cycle-associated genes [187]. YAP overactivation increases chromatin accessibility at TEAD motifs [188]. YAP activity correlates with CCCTC-binding factor (CTCF)-mediated chromatin reorganisation. It has been found that Hippo pathway activation results in both YAP and CTCF phosphorylation, which reduces the ability of CTCF to form chromatin loops [189]. Figure 3 describes the cytoplasmic and nuclear interactions of YAP/TAZ with signalling pathways, TFs, and chromatin-associated proteins.

### 5.3. YAP/TAZ/TEAD Interactions with TFs

#### 5.3.1. Activator Protein-1 (AP-1)

AP-1 is the second most abundant TF complex interacting with YAP/TAZ after TEADs. AP1 are dimers of the JUN (JUN, JUNB, JUND) and FOS (FOS, FOSB, FOSL1, and FOSL2) families of leucine-zipper proteins. AP-1 physically interacts with YAP/TAZ/TEAD, promoting target gene expression [184]. AP-1 binds up to 85% of enhancers involved in controlling YAP/TAZ target gene expression: TEADs and AP-1 occupy very similar regions [184]. AP-1 predominantly binds TEADs, although YAP and TAZ may also be involved in the organisation of the transcriptional complex via their WW and TB domains [190,191]. YAP-mediated chromatin reorganisation facilitates genome-wide AP-1 accessibility [188]. AP-1 has been identified as one of the primary factors that recruits SWI/SNF complexes [192]. YAP and TAZ could participate in this complex by binding ARID1A [105].

In the epidermis, AP-1 is one of the most critical TF complexes that regulate KC-specific gene expression and proper KC differentiation [193,194]. Interactions with AP-1 could be one of the key mechanisms by which YAP/TAZ/TEAD regulate the KC state.

#### 5.3.2. TEAD-Binding Proteins

Besides YAP and TAZ, VGLL1, VGLL2, VGLL3, VGLL4, and p160 can interact with TEADs to form transcription complexes [170]. The VGLL-TEAD interaction antagonises YAP-driven transcription in several cancer cell lines [195,196,197]. In the skin, VGLL4 inhibits YAP/TEAD-dependent transcription [197]; however, VGLL4 deficiency does not result in skin abnormalities [198]. Other VGLL proteins are associated with hyperproliferative phenotypes [199,200].

Members of the p160/SRC family, nuclear receptor coactivators 1 (NCOA1/SRC1), 2 (NCOA2/SRC2/TIF2/GRIP1), and 3 (NCOA3/SRC3/AIB1/ACTR), can interact with TEAD2 [201]. VGLL3 and p160/SRCs complexes are involved in skin immunity regulation and wound healing [202].

Since VGLLs and p160 antagonise YAP and TAZ through their ability to form complexes with TEADs and their expression is found in the skin under homeostasis and pathology, these proteins could be involved in modulating YAP/TAZ nuclear activity to control KCs’ fate.

#### 5.3.3. p63

p63 is a TF belonging to a p53 family involved in epidermal development, KC proliferation and differentiation, as well as apoptosis, stem cell maintenance, and adhesion [203,204] by activating and suppressing the expression of target genes, alongside mediating epigenetic regulation [204,205,206]. p63 has different isoforms created by alternative splicing: the TAp63 isoform has an N-terminal transcriptional activation (TA) domain, while dNp63 does not [207]. Both isoforms balance each other and act cooperatively during the stratification process [208,209]. dNp63 is predominantly expressed in the basal layer of the skin epidermis, stimulating cell proliferation and maintaining cell stemness [207]. TAp63 is involved in differentiation and stratification initiation [204].

In the epidermis, YAP/TAZ signalling and p63 are closely interconnected, mutually regulating each other [18,40,41,57,77,210]. dNp63α colocalises with activated nuclear YAP in proliferating basal KCs, while in dNp63α-negative suprabasal layers, YAP is located in the cytoplasm [41]. YAP and dNp63α act jointly to maintain the undifferentiated state of SCC cells [106,107,211,212].

YAP physically bound and stabilised dNp63 in HaCaT cells [77,213,214]. YAP and dNp63 could jointly regulate chromatin remodelling. YAP influences the accessibility of dNp63 target genes [215]. dNp63 interacts with the SWI/SNF complex, resulting in dNp63 target gene expression [206]. p63 cooperates in CTCF-mediated chromatin 3D structure reorganisation [216]. Since dNp63α and YAP/TAZ stimulate the expression of very similar targets and are involved in CTCF-mediated chromatin remodelling [189], this predisposes complex regulation of the availability of transcription regulatory elements in chromatin by YAP/TAZ and p63.

#### 5.3.4. Tumour Protein p73 (TP73)

TF p73 can interact with its homologues p53 and p63. Like p63, it is expressed in human KCs in vivo and in vitro [217]. Nuclear p73 can bind YAP through YAP’s WW domain and its poly-proline (PPPPY) motif [213,218]. YAP/p73 complexes are abundant in colon and breast cancer cell lines [218,219]; the YAP/p73 complex promoted apoptosis [77] and induced differentiation [220] in several cell types, the opposite effect of the traditionally mentioned YAP/TEAD complex. In epidermal KCs, YAP predominantly forms complexes with p63 rather than p73 [77], explaining the observed YAP-mediated effects on KCs.

#### 5.3.5. KLF Transcription Factor 4 (KLF4)

*KLF4* is a master gene regulating epidermal cell fate [221]. It regulates the transcription of epidermal differentiation genes involved in stratification and cornification [222]. The YAP/TAZ/TEAD complex negatively regulates *KLF4* expression. KC differentiation induced by TEADi requires KLF4, while *KLF4* KO increases transcriptional activity downstream of YAP1. KLF4 mediates YAP/TAZ/TEAD transcriptional activity via direct binding by its activation domain [68]. In cancer cells, KLF4 is suppressed, and YAP promotes snail family transcriptional repressor 2 (*SNAI2*) expression, which is involved in downregulating *KLF4* expression [74]. The interplay between KLF4 and the YAP/TAZ/TEAD complex regulates the balance between KC proliferation and differentiation.

#### 5.3.6. WW Domain Binding Protein 2 (WBP2)

WBP2 was recently identified as a TF connected to the YAP/TAZ/TEAD complex. WBP2 acts as a cofactor of YAP, enhancing TEAD-mediated transcription. WBP2 partially implements the YAP transcription programme. In SCC cells, WBP2 KD suppressed proliferation, while YAP KD also induced cell differentiation. WBP2 KO reduced basal KC proliferation and nuclear YAP accumulation during mouse embryogenesis [19].

#### 5.3.7. EMT-Related TFs

EMT is required for tissue regeneration and correct wound healing. It involves partial epithelial cell dedifferentiation, increased proliferation, and cell motility that enables the delivery of rapidly dividing epithelial progenitors to the injury site. After wound-closing, cells undergo redifferentiation and mesenchymal-to-epithelial transformation or cell senescence, followed by elimination by the immune system. Prolonged or chronic EMT disorganises the regeneration processes and arrests tissue architecture reconstruction due to extensive fibrosis or tumorigenesis [223]. YAP and TAZ behaviour follows all these principles: they are essential for correct wound healing and support cell proliferation and migration [14,84], with their overactivation in normal cells resulting in cell senescence under culture conditions [41], while YAP overactivation in mouse skin promoted tumour development with SCC characteristics [18,52]. YAP and TAZ do not promote cell de-differentiation [41] and thus only support EMT but do not induce it.

EMT is known to be driven by E-box DNA-binding TFs such as TWIST (mammalian twist family bHLH transcription factor 1 [TWIST1]), SNAIL (mammalian snail family transcriptional repressor 1 [SNAI1] and 2 [SNAI2/SLUG]), and Zfh1/2 (mammalian zinc finger E-box binding homeobox 1 [ZEB1] and 2 [ZEB2]). YAP signalling upregulates the expression of several EMT-associated TFs, including *ZEB1* [52,63] and *SNAI2* [63,74], and TAZ activation induces *TWIST1* [53]. YAP/ZEB1 nuclear binding enhanced tumour metastasis [224]. In mesenchymal cells, YAP/TAZ and SNAI1/2 can form nuclear complexes to maintain stromal cell renewal [224,225,226]. Therefore, these findings indicate that YAP/TAZ could cooperate with EMT TFs to promote mesenchymal cell fate and prevent EMT termination.

### 5.4. YAP/TAZ in LLPS

Recently, LLPS has emerged as an important mechanism of TF action [227,228]. Through LLPS, TFs form liquid condensates (droplets) that are highly dynamic and spherical, coalesce, and exhibit wetting behaviour. These condensates facilitate transcription, stabilise enhancer–promoter loops, and contribute to the specificity of transcriptional control, allowing its precise spatiotemporal regulation. LLPS depends on multivalent interactions between the unstructured parts of proteins, IDRs, and sometimes between other domains, such as CC or prion-like domains. Multiple TFs and core components of the transcriptional machinery contain IDRs and undergo LLPS [229,230].

Pioneering studies on YAP/TAZ LLPS have shown that TAZ-green fluorescent protein (GFP) and YAP-enhanced GFP (EGFP) form droplets in cells, the former only in the nucleus and the latter also in the cytoplasm [231,232]. Nuclear condensates of both YAP and TAZ concentrate the transcriptional machinery, open chromatin regions, and nascent RNA transcripts [231,233,234,235]. However, these studies relied heavily on the exogenous protein expression that could lead to overexpression artefacts [236]. Endogenous YAP and TAZ form puncta (foci in the nuclei) in cells, as revealed by immunofluorescence, which colocalise with nascent RNAs and depend on the same cues as YAP/TAZ-GFP droplets [231,232]. Further evidence that YAP/TAZ regulate transcription via LLPS comes from genetic engineering experiments. The CC and WW domains are both important for TAZ LLPS [165,232], whereas the IDR and CC domains are responsible for YAP LLPS [231,236]. Mutations in these domains abolish YAP/TAZ condensation in vitro. Consistently, these mutations disrupt endogenous YAP/TAZ puncta in cells and impair transcriptional regulation [231,232]. Notably, the IDR of YAP contains a pattern of positively charged amino acids, which appears to be essential for Mediator complex and RNA polymerase II (PolII) recruitment [237]. The Mediator complex and PolII attract additional YAP, facilitating condensate formation via a positive feedback loop [237]. The resulting condensates are further enhanced after treatment with transcription elongation inhibitor 5,6-dichloro-1-β-D-ribofuranosylbenzimidazole [237], indicating a possible antagonism between condensate growth and mRNA accumulation, consistent with previous observations [238]. This mechanism may provide a negative feedback loop for YAP condensation.

Hyperosmotic stress [231,239] and interferon-γ (IFNG) [236] induce YAP condensation in various cell types, which correlates with the upregulation of its target gene expression. However, YAP also forms smaller condensates in the nucleus under physiological conditions [233]. The number of such condensates depends on cell confluence and actin filaments. Interactions with TEAD proteins significantly enhance YAP condensation [233]. Single-particle tracking experiments conducted on YAP-TEAD condensates in vitro indicated that condensates slow YAP diffusion, possibly facilitating its target search [233]. Another consequence of YAP condensation is the formation of active transcriptional hubs [231,236]. In the ependymoma cells, the chimeric TFs YAP-mastermind-like domain-containing 1 (MAMLD1) and zinc finger translocation-associated (ZFTA/C11ORF95)-YAP form nuclear condensates and substantially reconfigure spatial genomic contacts as revealed by HiChIP analysis [240].

Unlike YAP, its paralog TAZ does not require crowding agents to undergo LLPS in vitro and forms liquid droplets in nuclei under normal conditions [232]. While TAZ is recruited into YAP condensates upon hyperosmotic treatment, a subset of genes are independently regulated by TAZ. The fact that TAZ more readily forms condensates than YAP may account for their regulatory specificity [232]. YAP/TAZ condensate formation is stimulated by matrix stiffness and actin filament polymerisation and is downregulated by the Hippo pathway [241]. Condensates also modulate TAZ activity through changes in viscoelasticity. The FUS RNA binding protein (FUS) associates with TAZ in nuclear condensates, maintaining their liquid state and thus facilitating TAZ-mediated transcriptional regulation [165]. When phosphorylated, FUS is no longer recruited to the TAZ condensates, resulting in their gelation and TAZ inactivation [165].

Despite the growing evidence that LLPS is essential for YAP/TAZ function, the quantitative contribution of their condensates to gene regulation remains an open question. Various drugs can modulate condensate formation. The most commonly used is 1,6-hexanediol (1,6-HD), which interferes with hydrophobic interactions [242]. However, YAP condensates are insensitive to 1,6-HD treatment [233], unlike TAZ condensates [165,232]. 1,6-HD also exhibits many side effects on chromatin architecture and cell viability [243,244,245]. Drugs such as VP, K-975, or peptide 17 can selectively dissolve YAP condensates by interfering with the YAP-TEAD interaction [233]. Nevertheless, developing strategies to specifically target LLPS without interfering with protein–protein interactions (as in mutation experiments) and general protein abundance (as in various depletion techniques) is crucial. Recently developed approaches that allow the on-demand recruitment of highly soluble proteins to condensates may be useful for further research [246,247,248]. These methods do not require any YAP/TAZ mutation or depletion while selectively disrupting condensate formation.

## 6. Conclusions

The functioning of YAP/TAZ in human skin under normal and pathological conditions is a cutting-edge area in investigative dermatology, embryology, and regenerative medicine, as these multifunctional proteins are associated with various skin diseases [41,45,46,47,48,49,50,51,52,53]. In addition, YAP and TAZ are paralogues; their functions are different in epidermis (Table 1). Published data indicate that YAP activation is associated with high proliferation and low differentiation independently in the IFE or HFs [40,42]. YAP is more important in homeostatic epidermal self-renewal than TAZ [67]. In addition, YAP acts with TAZ in epidermal injured phenotype formation [14,65,66,84].

**Figure 4:**
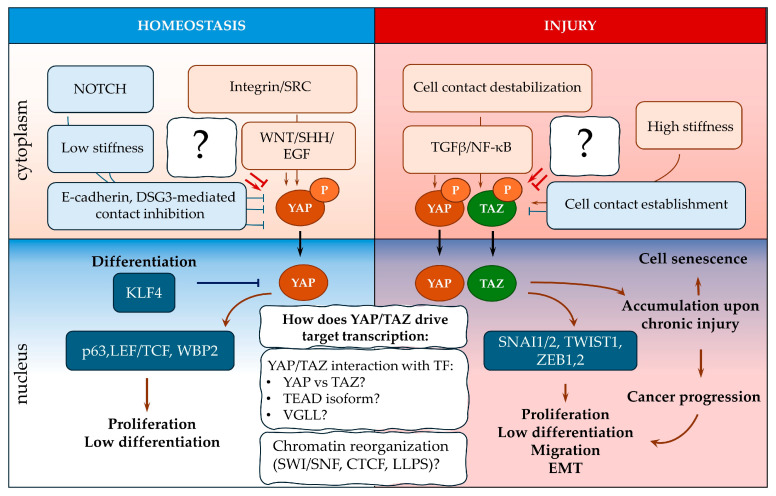
Functioning of YAP/TAZ in the epidermis under normal and pathological conditions. Several aspects of the functioning of the YAP/TAZ currently require further investigation: the influence of VGLL on KC differentiation; the role of different TEAD isoforms in the expression of YAP/TAZ target genes; and how the organisation of protein complexes and reorganisation of chromatin structure regulate transcriptional activity.

The nuclear activity of YAP and TAZ should be transient for proper skin physiology. Many mechanisms can inactivate YAP/TAZ and sequester them in the cytoplasm, including the Hippo pathway and contact inhibition during proliferation [18,19,40,61,97,98]. YAP activation in basal KCs is mediated via organisation cell–matrix contacts with the basal lamina by integrin and subsequent SRC activation [14,90]. YAP cooperates with the WNT, SHH, and EGF pathways and p63, SNAI2, and WBP2 TFs to prevent differentiation and stimulate basal KC proliferation [14,18,19,21,22,23,40,41,57,77,79,82,98,210]. Furthermore, YAP must be inhibited. The possible YAP-induced decrease in COL17A1 in KCs [61] results in the loss of adhesiveness with the basal membrane, their movement to the suprabasal layers with decreased microenvironment stiffness. Excluding KCs from the epidermal stem cell niche decreases WNT and SHH levels. These conditions suppress YAP activity; thus, YAP is cytoplasmic in spinous KCs and absent in superficial layers. KLF4 and NOTCH, which are inhibited by YAP, stimulate KC differentiation [59,68,74,126] (Figure 4).

During wound healing, YAP and TAZ are insufficient for partial KC EMT but support it by promoting proliferation and inhibiting differentiation [43]. During injury, YAP/TAZ activation is mediated by escaping contact inhibition due to epidermis disruption and mechanotransduction due to increased matrix stiffness in the wound [59,98,101,104]; TGFβ, EGF, FGF, and other signalling pathways are also involved [23,24,25,44,53,131,132]. YAP/TAZ interact with EMT-associated TFs and stimulate KCs to initiate the inflammation process, including KRT6/16/17 expression, dedifferentiation, migration, and synthesis of cytokines that attract immune cells [52,63,74,224]. YAP and TAZ activity must be arrested after the regeneration process is complete. Establishing AJs and subsequent contact inhibition could suppress YAP/TAZ or, under prolonged activation, could upregulate the expression of cell senescence markers, leading to the removal of cells from the tissue by immune cells [41]. Chronic inflammation leads to the accumulation of YAP and TAZ in nuclei, causing hyperproliferation of the epidermis in several skin diseases [45,46,47,48]. Tumour formation is also often linked to the dysregulation of YAP/TAZ inhibition mechanisms [49,50,51,52,53] (Figure 4).

YAP/TAZ regulate KC phenotype-specific gene expression by creating protein complexes at enhancers and super-enhancers and reorganising chromatin structure to upregulate the availability of DNA sites for TF binding. YAP and TAZ primarily interact with TEADs [170] and AP-1 [184], which directly binds DNA, to promote target gene expression by recruiting BRD4/Mediator [185] and MMB complexes [187]. YAP cooperates with β-catenin/LEF/TCF and p63 to upregulate stemness genes in homeostatic skin [41,122,123] and with TAZ, SMAD2/3, SNAI1/2, and ZEB1/2 in the injury response [52,63,74]. Under chronic inflammation or tumour progression, accumulated nuclear TAZ could stabilise the complexes between YAP/TAZ and cancer-associated TFs by forming condensates via LLPS [232], thus promoting cell senescence escape and cancer therapy resistance. KLF4 suppresses the YAP/TAZ-mediated transcription machinery and induces KC differentiation. VGLLs are also predicted to participate in YAP/TAZ protein complexes; however, their influence on KC differentiation must be explored. Similarly, studies must determine the roles of TEAD isoforms in YAP/TAZ-mediated target gene expression, particularly since the formation of different TFs with YAP/TAZ can provoke different effects in epidermal skin cells under different conditions. The ability of YAP and TAZ to create molecular condensates via LLPS highlights a gap in understanding how the organisation of protein complexes regulates transcription activity. Understanding the specific details of the mechanisms of YAP/TAZ activation and suppression and their transcription promotion in epidermal KCs would support drug development for hyperproliferative epidermal diseases and anti-cancer therapy, as well as for maintaining KC proliferation and stimulating wound healing in epidermolysis bullosa or chronic ischemic wounds due to diabetes.

## Figures and Tables

**Table 1:** Diversity in YAP and TAZ functions in epidermis.

Process	YAP	TAZ
Homeostasis		
Proliferation	↑ (in vivo/in vitro)	↑ (only in vitro)
Differentiation	↓	-
Injury		
Migration	↑	↑
Proliferation	↑	↑
EMT	↑	↑

↑/↓ indicate stimulation/suppression of processes by YAP or TAZ.
